# Real-time MRI: a new tool of radiologic imaging in small children

**DOI:** 10.1007/s00431-023-04996-0

**Published:** 2023-05-30

**Authors:** Franz Wolfgang Hirsch, Jens Frahm, Ina Sorge, Dirk Klee, Freerk Prenzel, Matthias Krause, Martin Lacher, Dirk Voit, Daniel Gräfe

**Affiliations:** 1 Department of Pediatric Radiology, University Hospital, Leipzig, Germany; 2 Biomedical NMR, Max Planck Institute for Multidisciplinary Sciences, Gottingen, Germany; 3 Department of Radiology, University Hospital, Dusseldorf, Germany; 4 Department of Pediatrics, University Hospital, Leipzig, Germany; 5 Department of Neurosurgery, University Hospital, Leipzig, Germany; 6 Department of Pediatrics Surgery, University Hospital, Leipzig, Germany

**Keywords:** Magnetic resonance imaging, Anesthesiologists, Workflow, Radiology, Pediatric radiology

## Abstract

Real-time MRI (rt-MRI) in children is a new imaging technique that combines the advantages of US — at frame rates of up to 50 images per second — with the quality and features of MRI. Although still subject of research, it has become a standard tool in the diagnostic portfolio of two pediatric radiology departments in Germany. Based on ultrashort acquisition times, any detrimental effects of macroscopic movements of the child and the physiological movements of the organs are negligible. Especially in pediatric brain imaging, rt-MRI has already proven its value. With suitable indications, rt-MRI can reduce anesthesia and sedation examinations in children below 6 years of age by 40% due to its very short examination time and its robustness to motion. There is a high level of acceptance among parents and referrers when diagnostic possibilities and limitations are communicated correctly.

*Conclusion*: Completely new diagnostic possibilities arise in the imaging of the moving lung, the beating heart, joint movements, and speaking and swallowing, as demonstrated in this video-backed review.

**Table:** 

**What is known:** • *MRI in moving children has been burdened with severe artifacts.* • *Gross motion usually has to be handled by sedation and periodic motion of the heart and lungs has to be compensated with time-consuming techniques until now.*	
**What is new:** • *Real-time MRI allows image acquisition with up to 50 frames per second similar to ultrasound frame rate.* • *Real-time MRI proofs to be very promising for imaging children, reducing examination time and sedation rate drastically.*

**Supplementary Information:**

The online version contains supplementary material available at 10.1007/s00431-023-04996-0.

## Introduction

Real-time MRI (rt-MRI) with ultrafast image acquisitions of down to 20 ms (50 images per second) is an entirely new approach in pediatric radiology. Although the technique was described 13 years ago [1], rt-MRI was first applied in pediatric imaging only 3 years ago. This is remarkable because the resistance to movement renders rt-MRI an obvious choice in small children below 6 years of age. Each individual image is generated so quickly that every motion is frozen.

This rt-MRI technique was developed by the Max Planck Institute in Göttingen/Germany, initially for cardiac imaging [1, 2]. Scientific reports applying this method to children have been published since 2020 [3–5]. Therefore, it seems necessary to promote this new technique not only within the community of pediatric radiologists but also to referring colleagues in pediatrics and pediatric surgery. In this review, the current possibilities, and limitations, and illustrative videos of typical rt-MRI applications are presented to pediatricians and pediatric surgeons.

## Faster than the child’s movement and the movement of the heart and lungs

Every type of motion, whether the gross movement of the patient or periodic motion such as breathing or heartbeat, is a well-known hurdle to conventional MRI, even with conventional fast MRI sequences of up to 5–10 frames per second (fps). The requirement to lie still is a major obstacle to the examination of younger children. In contrast, ultrasound (US) had a decisive advantage: due to the fast image frame rate of about 25 fps, US always produces sharp images, even in moving organs or uncooperative patients. However, several limitations of US have been well noted: The basic requirement for US is invariably a suitable sound window. As reflections occur at bone and air interfaces, diagnosis around those structures is often hindered. In addition, only a small part of the body can be examined simultaneously, and, most importantly, there is a strong dependence on the examiner and very few possibilities for post-hoc assessment of US images [6].

MRI with a comparable temporal resolution as US has so far failed due to apparent physical limitations. Therefore, “conventional” MRI, with an acquisition time of at least 100 ms even in fast MR sequences, remains very susceptible to periodic movements of the organs and to spontaneous macro-movements of the child. Whereas for the former, time-consuming mechanisms for compensation are available, the latter to date often still requires sedation or anesthesia in children under 6 years of age [7].

Only recently, a breakthrough in ultrafast MR imaging has been made with acquisition times of down to 20 ms for a single image [8]. With this novel approach to data sampling and subsequent image reconstruction, the image repetition rate of these MRI sequences (50 fps) is even faster than with US (around 25 fps). Therefore, this new MR technology is referred to as rt-MRI. Although other rt-MRI techniques have their own advantages and disadvantages, the following review refers to a specific rt-MRI method, also called FLASH 2.0. RT-MRI paves the way for a whole new range of applications for MRI. Three core strategies for rt-MRI have been identified:

- Continuous ultrafast image acquisition remains on a single slice (e.g., on the heart or a joint) to directly observe motion in a cinematic fashion.
- Continuous ultrafast image acquisition of a slice and continuous shifting of this slice to scan an entire volume (e.g., the chest) in a very short time with artefact-free individual images, named “volume coverage” (VC) technique.
- Continuous ultrafast image acquisition remains on a single slice with interactive, real-time manipulation of the slice position and orientation with visual control.

## A brief explanation of the real-time MRI technique

Without discussing the physical details of real-time MRI in depth, the technical principles can be summarized as follows:

This technique combines highly undersampled radial *k*-space acquisitions in conjunction with a joint reconstruction of images and coil sensitivity maps by nonlinear inversion with temporal regularization to the preceding frame. Unlike conventional MRI sequences, rt-MRI image reconstruction does not rely on Fourier transformation but instead uses an iterative reconstruction algorithm that exploits the similarity of successive frames. This approach allows image reconstruction with a sampling of only 2–4% of the complete *k*-space data and thus achieves a corresponding 25–50-fold reduction of the measuring time [9].

A simple comparison can illustrate the progress in terms of image acquisition since the beginning of medical MRI: While in 1985, the acquisition of a single MRI slice still took about 5 min; rt-MRI accelerates image acquisition by 15,000-fold. With this speed, rt-MRI invades a domain that was previously occupied by “real-time” US. How can children benefit from this new approach to MRI? Which applications have been established yet? What are the limitations of rt-MRI?

## Morphological imaging of organs

In most situations, the pediatrician or pediatric surgeon is interested in morphological imaging of organs to confirm or exclude a suspected pathology. To fulfill this task without sedation in small children, various fast techniques have been reported. However, concerning the speed of acquisition, they do not come close to rt-MRI [10, 11].

**Fig. 1:**
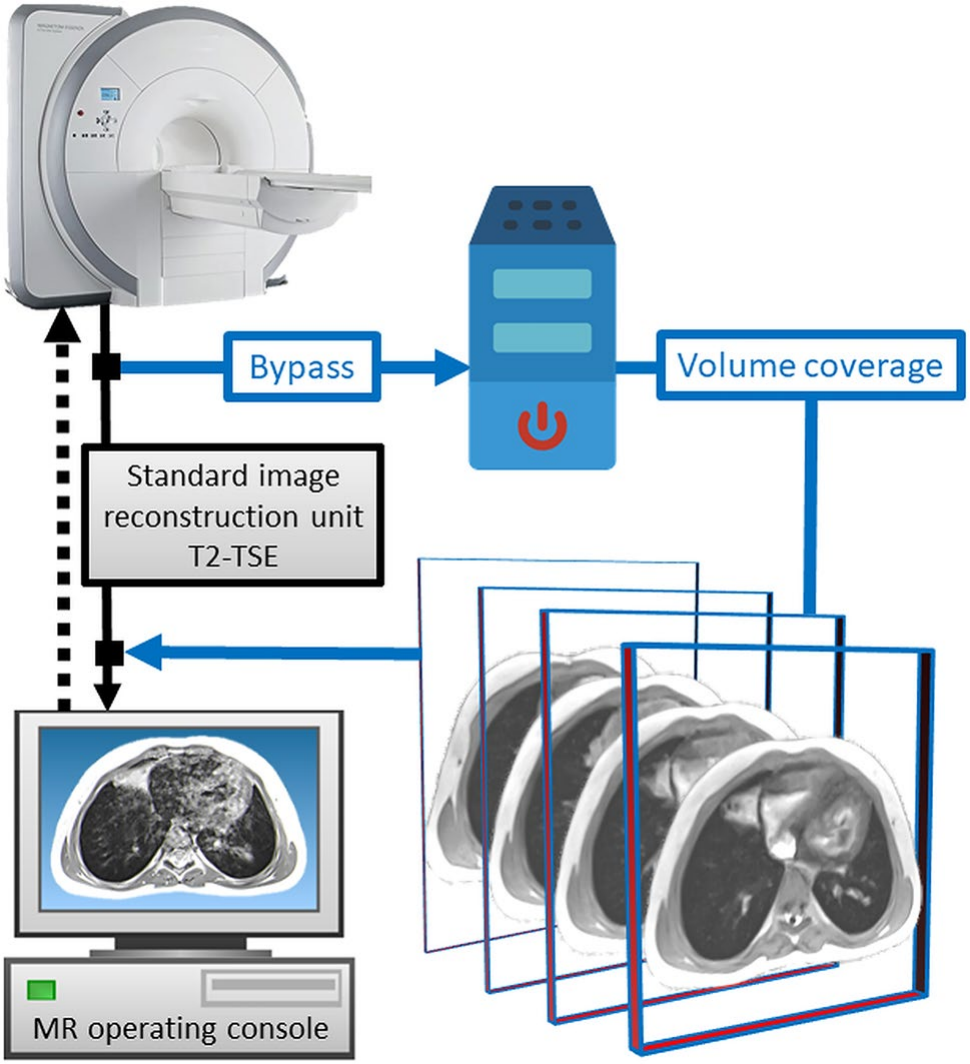
Schematic illustration of a vendor-neutral data flow for generating real-time MR images using the volume coverage sequence as an example (blue pathway). The real-time technology integrates seamlessly into the standard MR workstation workflow like any genuine, conventional MR sequence [20]

For imaging of an entire organ, a specific variation of rt-MRI called “volume coverage” is most often employed (Fig. 1) [12, 13]. The following applications of the VC technique have already widely been transferred in daily routine in our department of pediatric radiology. Although, in principle, every gradient-recalled echo sequence scheme may be used in rt-MRI, two weighting schemes for the VC sequence are most used: proton-density weighting (PD) and T2/T1 weighting [4].

## Brain examinations of infants with volume coverage technique

The VC technique enables the scanning of a large volume, such as the brain, the lung, or the abdomen, within a few seconds. Herein, numerous 2D-slices are acquired, and each of these slices is free of motion artifacts due to a per-slice acquisition time of down to 20 ms [13]. Thus, even with significant macro movements of the child, free breathing and conscious MRI are possible. In our department, parents are placed together with the child in the MRI bore. They gently fix the hearing protection of their child with the flat of their hands; strict fixation is unnecessary (Video 1). The playback speed of all videos presented in this review corresponds to the actual duration of the data acquisition.

**Figure:**
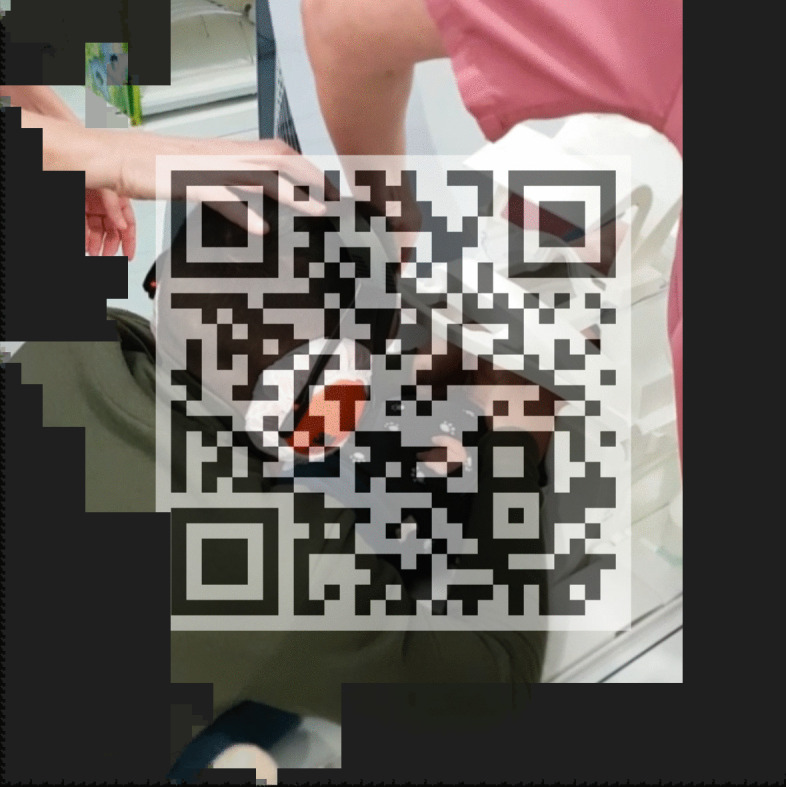
Video 1 Video of a real-time MRI in a non-sedated infant in our department. The father is placed together with the child in the scanner bore of the MRI. He gently supports the patient’s head with the flat of his hands. Remaining movements of the child do not interfere with imaging because of the ultrafast acquisition time down to 20 ms per image

In VC, with every new image, a tiny slice shift is introduced. This results in a large number of slices with a high overlap of 50 to 90%. With typical parameters, the acquisition of 300 slices at 3 mm width and a slice shift of 0.3 mm takes about 15 s. Thus, the steady state of proton excitation is maintained, which results in favorable tissue contrasts. In addition, the overlapping slices create a harmonious, gliding image impression. Despite the minimal slice thickness of several millimeters needed in 2D MRI, the image impression equals that of a 3D sequence.

For the radiographer, there is no change to his general workflow at the MR console. The rt-MRI sequences are planned and performed “out of the box” like any genuine, conventional MR sequence (Fig. 1). After data acquisition, the images are displayed directly on the workstation of the MR scanner or on any Picture Archiving and Communication System workstation. Though the rt-MRI technique is compatible with any MRI scanner, the user interface is currently adapted only for Siemens MR scanners (Erlangen, Germany).

After almost 3 years of routine use, we conclude that this new, fast type of MRI addresses an obvious need among our clinical colleagues. In brain imaging after fontanel closure, rt-MRI with VC is often needed for alterations of the cerebrospinal fluid (CSF) spaces and for the exclusion of gross pathology. However, apart from some specific situations such as minimal handling in very low birth weight preterm children, rt-MRI, when available, is often preferred over the US examination, even when the fontanel is still open, especially when surgical planning depends on imaging. Unlike US, rt-MRI does not require an US window such as the anterior fontanels and thus provides a complete overview of the entire brain, including the brain stem and the cerebellum. Additionally, rt-MRI is often faster and provides a superior soft tissue contrast than US.

Regarding CSF spaces, VC has been proven to be equivalent to a conventional T2-fast spin-echo sequence in small children but with considerably fewer motion artifacts [3]. Beyond CSF spaces, rt-MRT also seems suitable for other indications like trauma and unclear disturbances of consciousness in small children.

With the example of three typical findings, hygroma, hemorrhage, and tumor (Videos 2, 3, and 4), the reader can get an idea of the diagnostic potential of cerebral rt-MRI with VC. Cerebral rt-MRI has been established as a modality of choice in our department for urgent confirmation or exclusion of gross pathology (e.g., tumors or acute hemorrhage) in small children without sedation. In case of pathologic findings, a dedicated conventional MRI is subsequently scheduled under sedation by a pediatric anesthesiologist. Regarding small children below 6 years of age, the introduction of rt-MRI for evaluation of CSF spaces, as in children with hydrocephalus, has led to a 40% reduction in the proportion of brain MRIs under sedation in our department [14].

**Figure:**
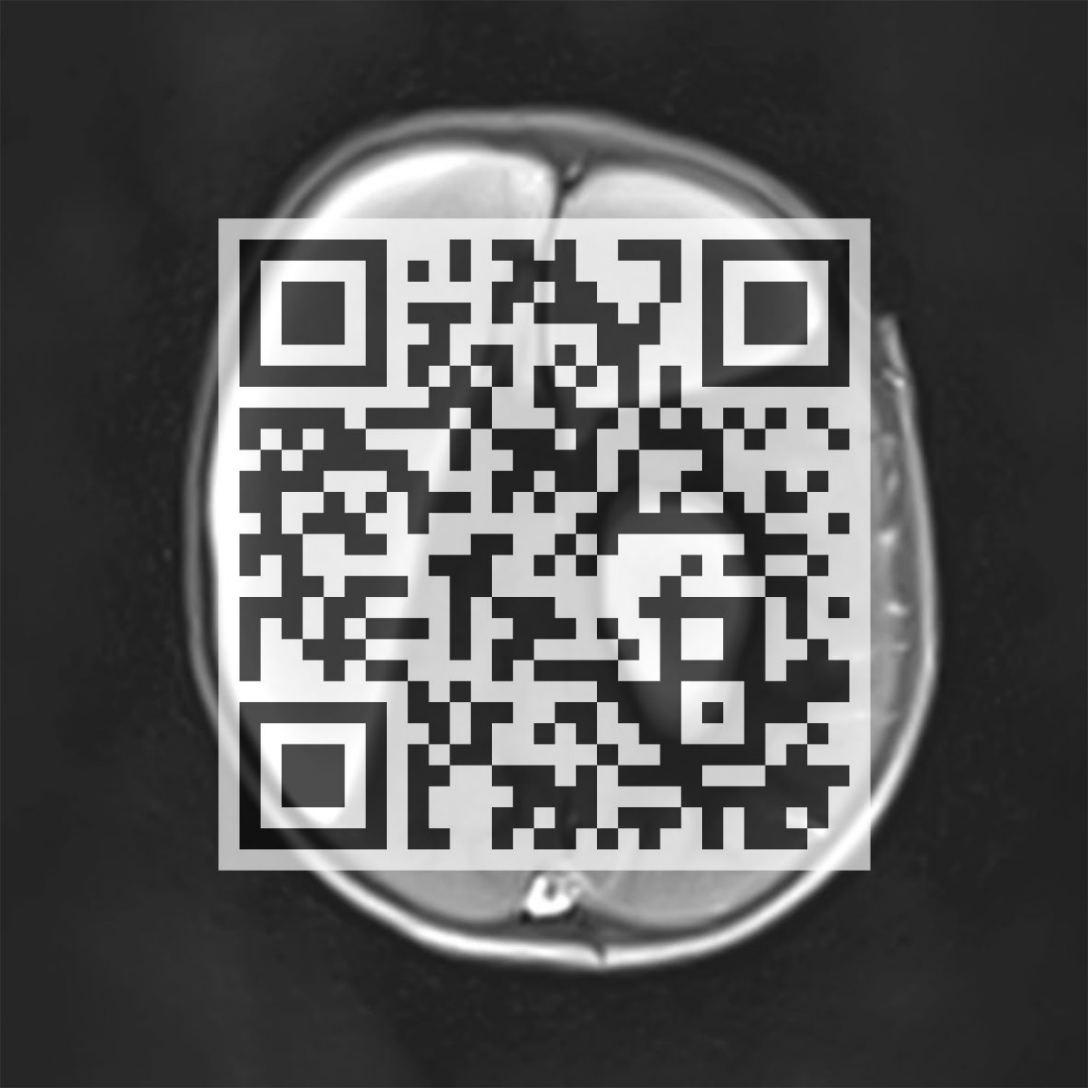
Video 2 Brain volume coverage (T2/T1 weighted) of a 3-year-old boy with a malformation in the frontal brain. The anterior horns of the lateral ventricles are not covered by parenchyma until they reach the rostral skull. A subdural hygroma has developed on the right side. On the left side, a narrow membrane separates the enlarged left lateral ventricle from the external subarachnoid space (red asterisk). Additionally, there is a shunt catheter with a tip in the right subarachnoid space. This patient underwent a total of 21 brain MRIs due to relapsing shunt dysfunction. 18 of the examinations have been performed without anesthesia by real-time MRI, only by gentle fixation of the child by a parent

**Figure:**
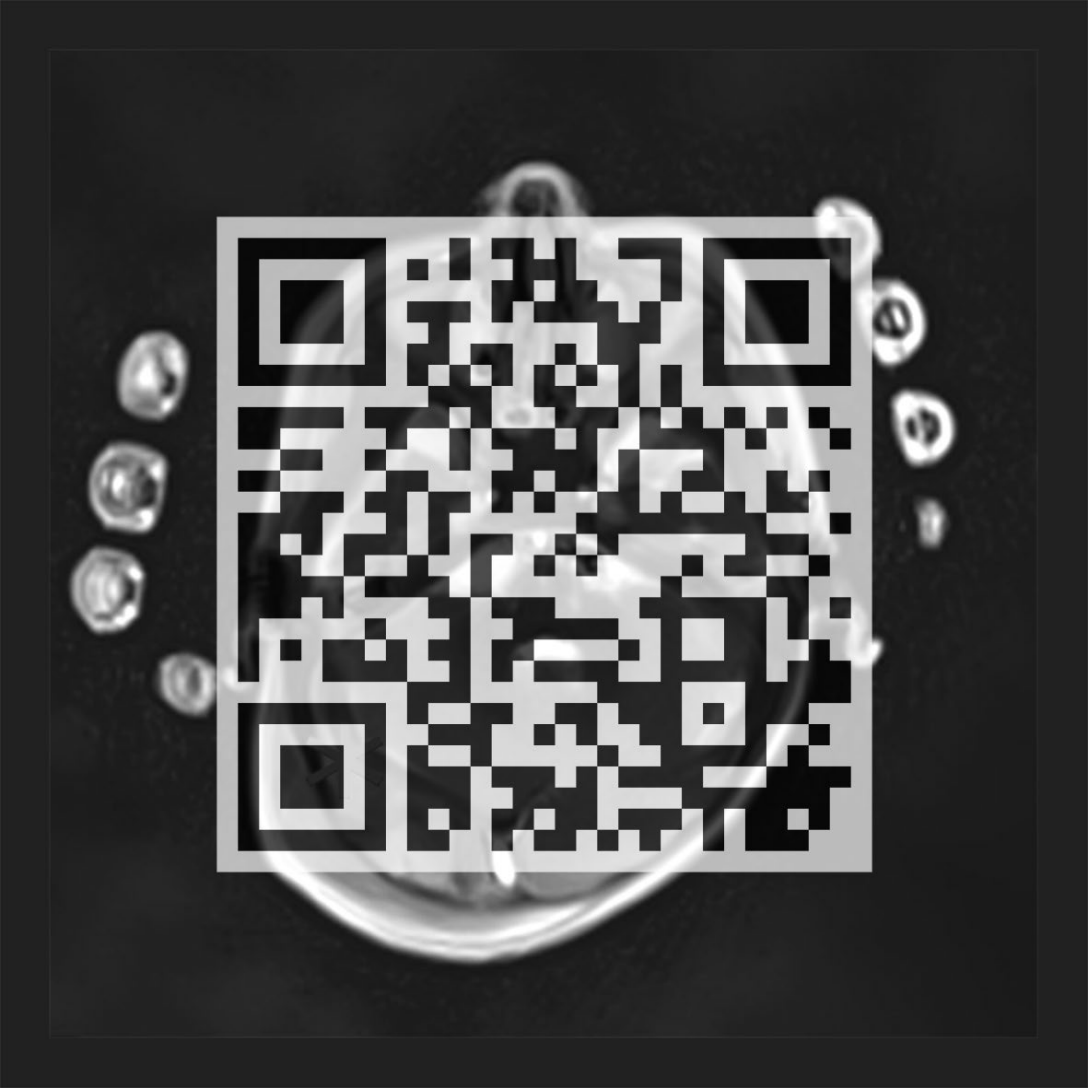
Video 3 A 14-month-old boy after falling from his mother’s shoulder four hours ago. The patient had a right occipital skull fracture with epidural and intraparenchymatous hemorrhage (red asterisk) in brain volume coverage sequence (T2/T1 weighted)

**Figure:**
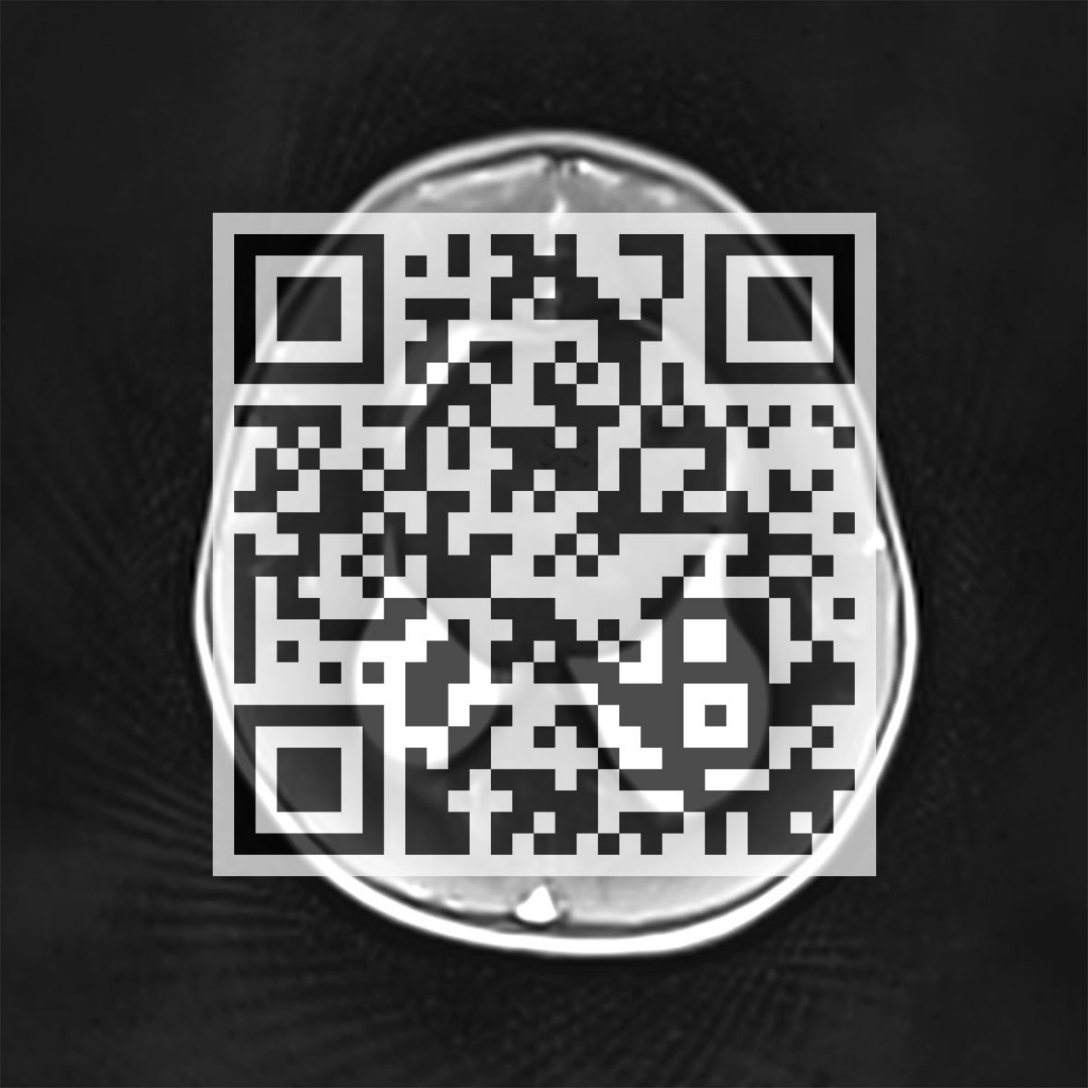
Video 4 An 8-month-old girl with fever, increased C-reactive protein, and neck stiffness. Rt-MRI (T2/T1 weighted) was requested to rule out elevated intracranial pressure prior to the spinal tap. A large mass in the midline with extension into both lateral ventricles is shown (red asterisk) with consecutively impaired liquor drainage, as evident on the ballooned temporal horns (histology: pilocytic astrocytoma)

## Observing the beating heart

Ultrafast acquisition in the same slice position enables the visualization of periodic or non-periodic motion in real-time [15]. Regarding cardiac imaging, rt-MRI, unlike conventional MRI, is fast enough to depict the beating heart in real-time with a sufficient temporal and spatial resolution [16]. This application is especially valuable in small children who cannot perform breath-hold maneuvers or children with arrhythmia when electrocardiogram gating does not yield decent results [5] (Video 5).

**Figure:**
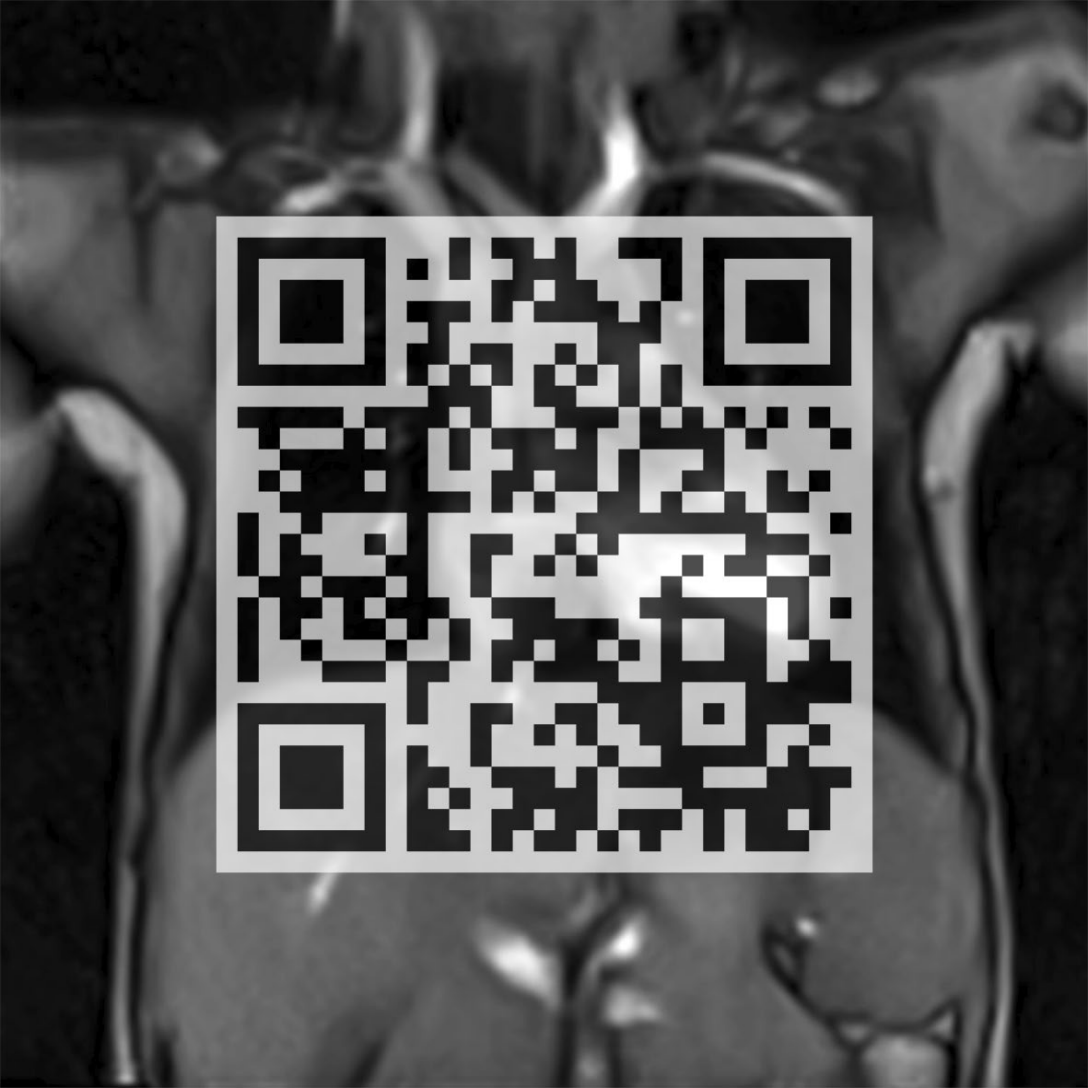
Video 5 Cardiac imaging (proton density weighted) of a 12-year-old boy healthy volunteer in free breathing in coronal orientation with a temporal resolution of 30 images per second

## Blood flow and cerebrospinal fluid movement in real time

Due to the inflow effects of blood and CSF in gradient-recalled echo sequences, rt-MRI is well suited for the non-contrast imaging of medium to large blood vessels [17]. In contrast to conventional static MRI, the intensity of the vessel varies periodically with the pulse wave. Therefore, rt-MRI proved helpful, for example, in assessing the interaction of the aortic arch and the supra-aortic vessels with the trachea (Video 6).

**Figure:**
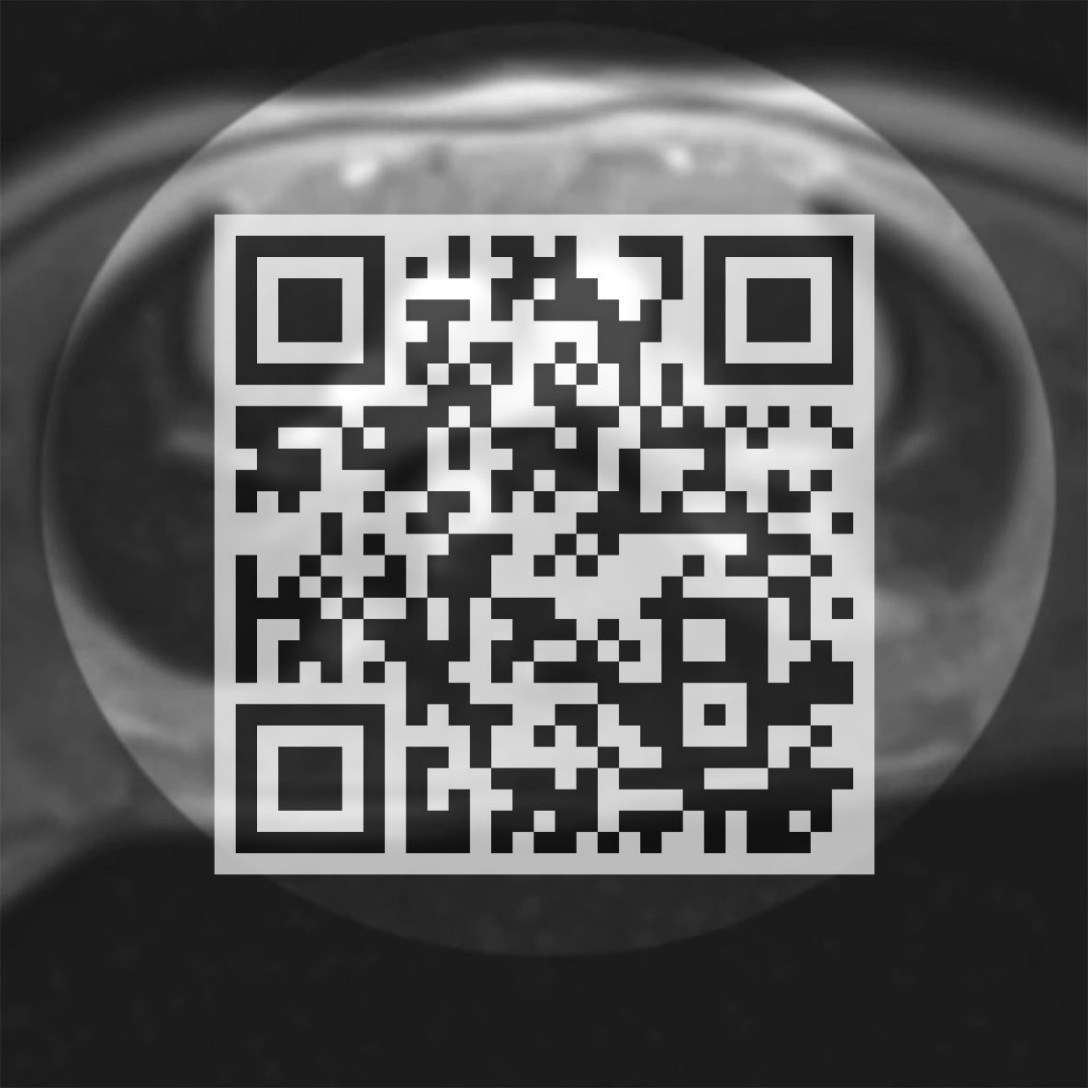
Video 6 A 1.5-year-old boy with stridor. There is a narrow region of the trachea directly behind the pulsating brachiocephalic trunk and straight in front of an aberrant left subclavian artery. Together with the clinical symptoms, this finding is suspicious of an artery compression syndrome. Due to the inflow effect, the vessels display a high signal even in the absence of a contrast medium (proton density weighted MRI)

## Watching the lungs during breathing

There is an obvious potential for real-time imaging of the lungs during free breathing. Periodic motion due to breathing and the heartbeat hinders conventional thorax diagnostics in MRI as it causes artifacts. Though periodic motion can be quite well compensated by respiratory gating, breath-hold maneuvers, and ECG-gating, those mechanisms prolong the examination time considerably. Furthermore, they are dependent on a consistent periodic pattern and fail in cases of irregular breathing or arrhythmia [18]. This is not the case with rt-MRI, which allows you to directly observe the lungs while they are breathing. This is a unique aspect compared to even computed tomography (CT) as the standard of thoracic cross-sectional imaging. Furthermore, it allows dynamic examination of the diaphragm and diaphragmatic defects [19].

The visual impression of the lung rt-MRI resembles a CT scan. Often, VC sequences, as noted above, are employed. Coronal orientation for acquisition is preferred to preserve the steady state of the lesions during the predominantly head-feet motion during the breathing cycle. Although T2/T1 contrast should, in theory, result in a superior lesion-to-background contrast compared with T1 or PD-weighted imaging, this advantage is offset by the pronounced fluctuation of signal intensities caused by the loss of the steady state in through-plane motion. Therefore, both are often performed with the more stable proton-density weighting and the contrast-rich T2/T1 weighting. Even small lesions, such as metastases, can often be detected above a diameter of 4 mm (Video 7), but only lesions larger than 10 mm can be depicted securely [20]. Therefore, rt-MRI of the lungs is not yet ready for screening for small metastases. In contrast, pneumonic infiltrates, abscesses, and pleural effusions can be readily detected (Video 8). We consider rt-MRI to be superior to X-ray and US in this regard.

**Figure:**
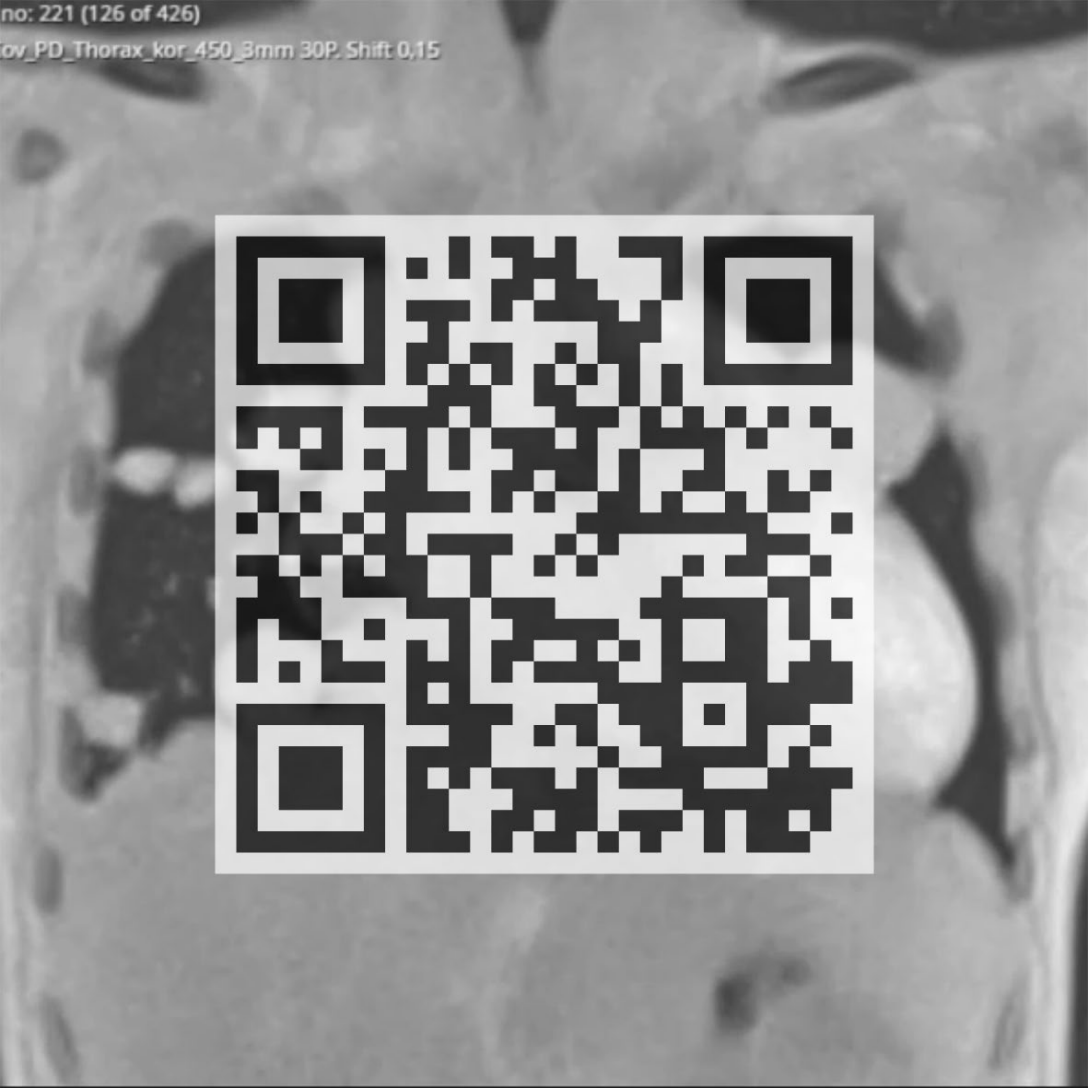
**Video 7** Proton-density weighted volume coverage sequence in coronal orientation (video on the right side) in a 14-year-old girl with multiple lung metastases from sarcoma in the lower leg in comparison with the x-ray (on the left side)

**Figure:**
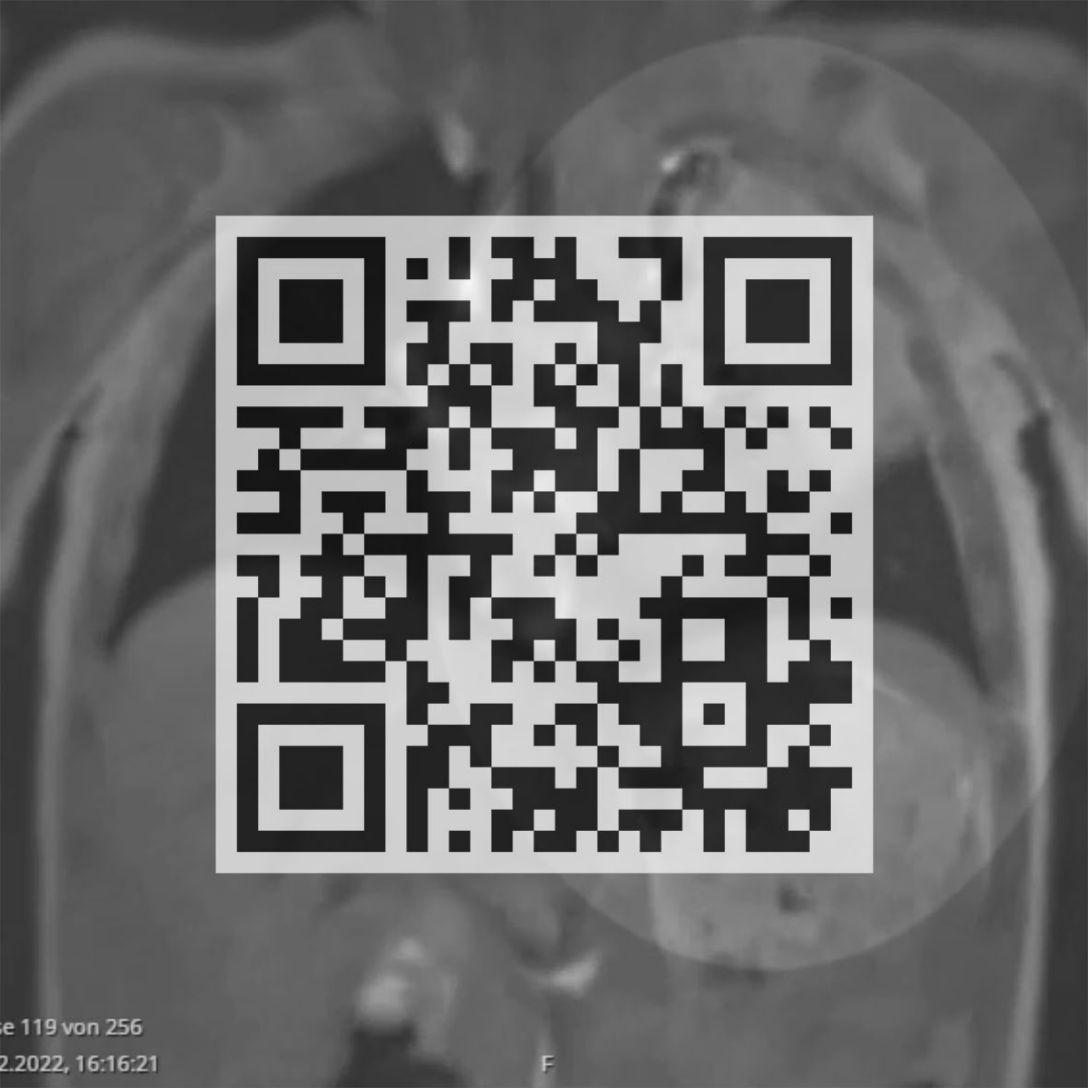
Video 8 T2 /T1 weighted volume coverage sequence in coronal orientation (video on the right side) in a 1.5 -year-old boy with a pneumonic consolidation in comparison with the x-ray (on the left side). There are no motion artifacts despite the free breathing of the patient. Hence the infiltration in the left upper lobe is well depicted. In contrast to the X-ray, a small abscess can be seen within the pneumonia by real-time MRI (bright signal, red arrow)

## Visualizing speaking and swallowing

The movement of the tongue and soft palate are easily visualized with rt-MRI [21, 22]. In children with cleft palates, dynamic evaluation of the soft palate closure during speech delivers important additional information to the maxillofacial surgeon regarding concomitant velopharyngeal insufficiency (personal communication). Under observation in rt-MRI, a defined set of words is pronounced, and the occlusion of the soft palate on the posterior pharyngeal wall and the mobility of the soft palate are assessed (Video 9).

**Figure:**
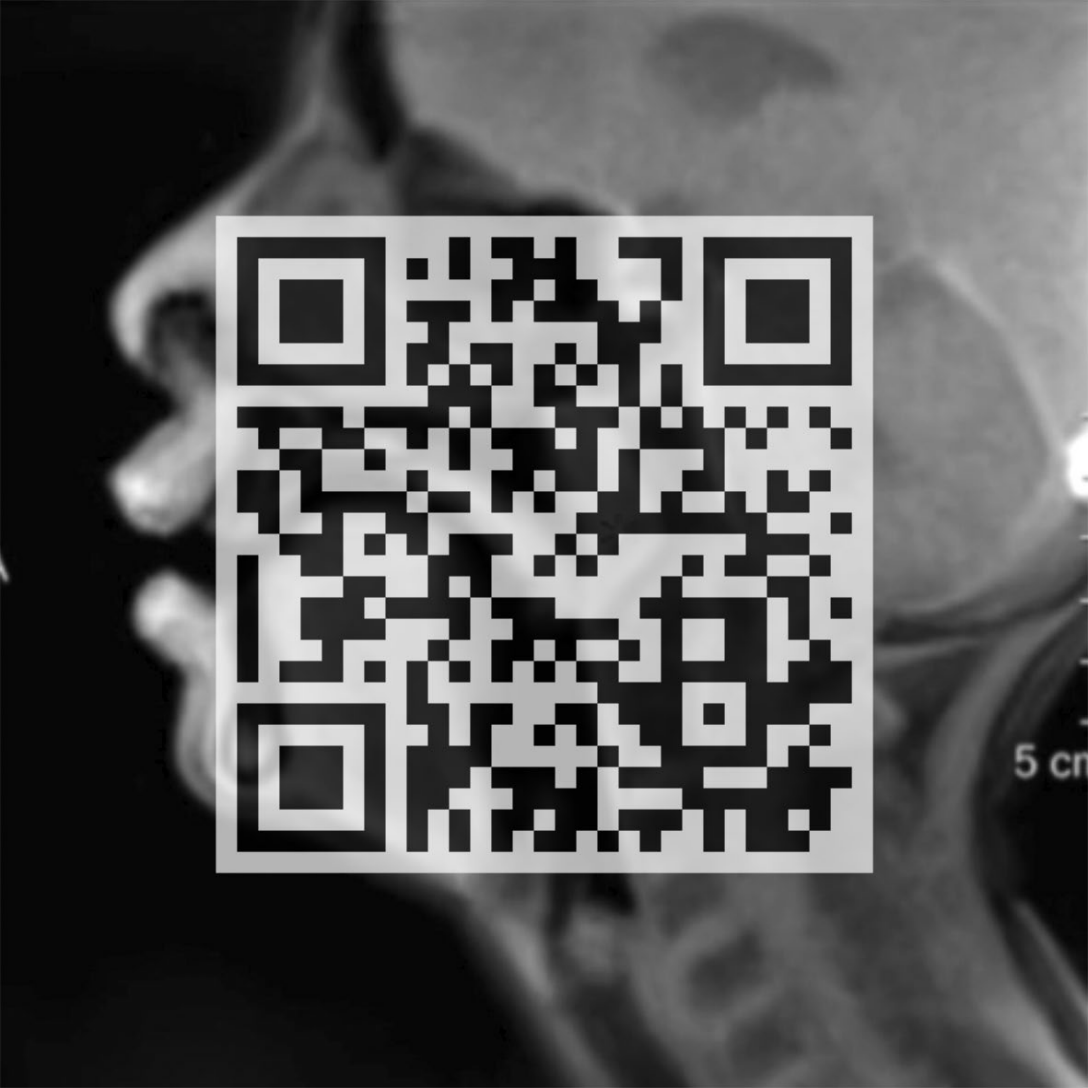
Video 9 Real-time MRI (proton density weighted) of the pharynx of a 5-year-old girl after surgery for a mucous cleft palate. While speaking the word “crocodile,” closure of the nasopharynx cannot be observed. The soft palate was surgically fixed to the posterior pharyngeal wall (red asterisk) and is therefore permanently attached here [4]

Functional upper GI tract diagnostics, including swallowing, tests for gastroesophageal reflux, and gastroduodenal transport, can be evaluated in adults using rt-MRI [23–25]. However, a greater extent of patient compliance is required: the patient must suck in a drink with a T1-hyperintense signal (such as pressed pineapple juice or diluted gadolinium) in a supine position within the scanner bore. The contrast medium must not be swallowed until the MRI is ready. Also, in children, rt-MRI may be an alternative to the fluoroscopic examination (Video 10). Yet, due to the complexity of the procedure, this method appears to be reserved for children above ten years. In younger children, we would currently still prefer fluoroscopic imaging, especially when aspiration of contrast media must be expected.

**Figure:**
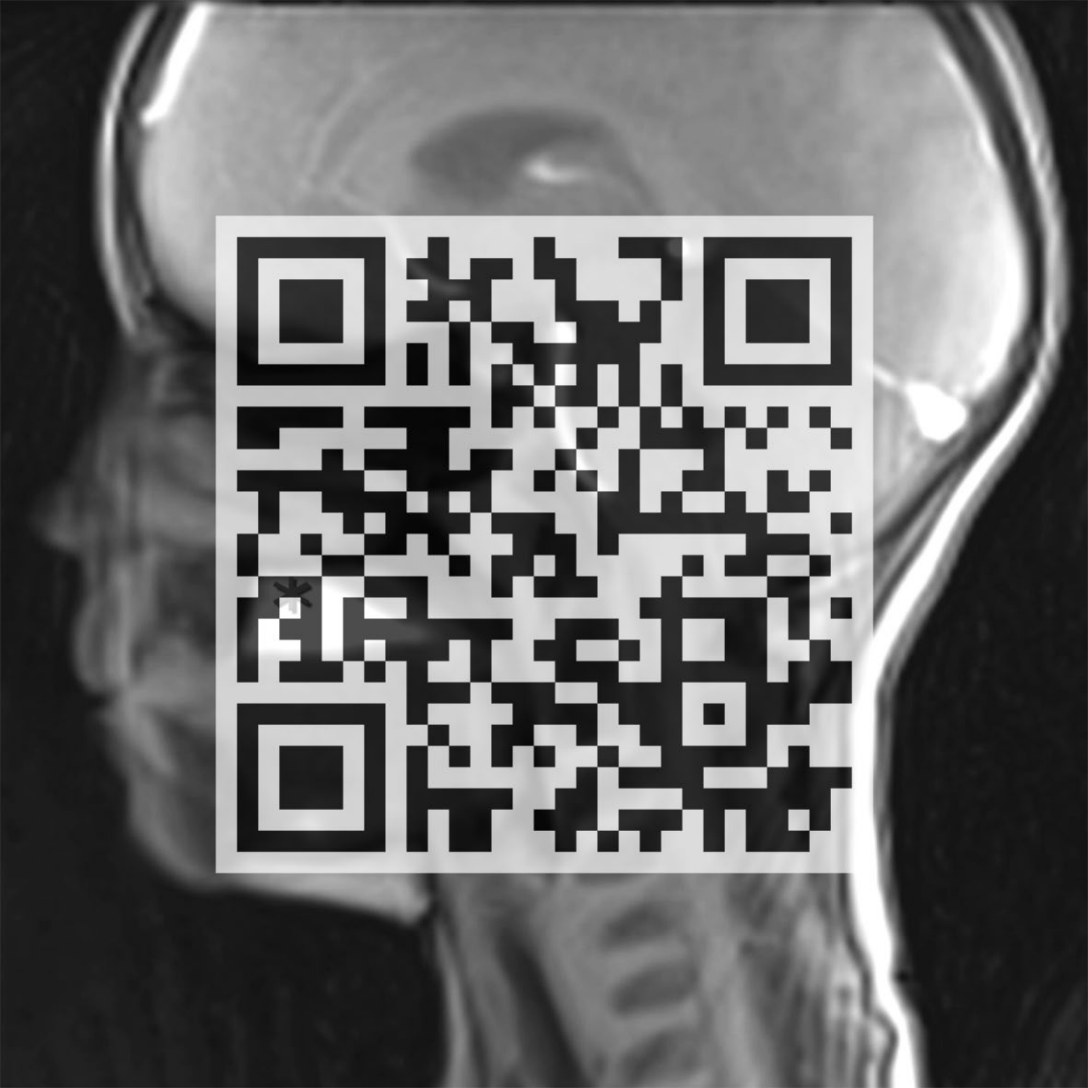
Video 10 10-year-old girl with suspected dysphagia. The swallowing act (proton density weighted) shows a regular pharyngeal passage. Pineapple juice (red asterisk) was used as contrast media

## Dynamic joint examinations and static scoliosis imaging

Imaging of joint movement with rt-MRI has previously been shown in adults, focusing on the temporomandibular joint and the knee [26, 27].

Rt-MRI was used to dynamically assess the upper cervical spine, e.g., in children with suspected atlantoaxial instability (Video 11). The changing distance in the atlantoaxial distance can be visualized more precisely in rt-MRI than in conventional static MR. It appears even superior to fluoroscopy because, in the latter, the position of the dens axis is sometimes more difficult to assess due to the superimposition of the atlas arch.

**Figure:**
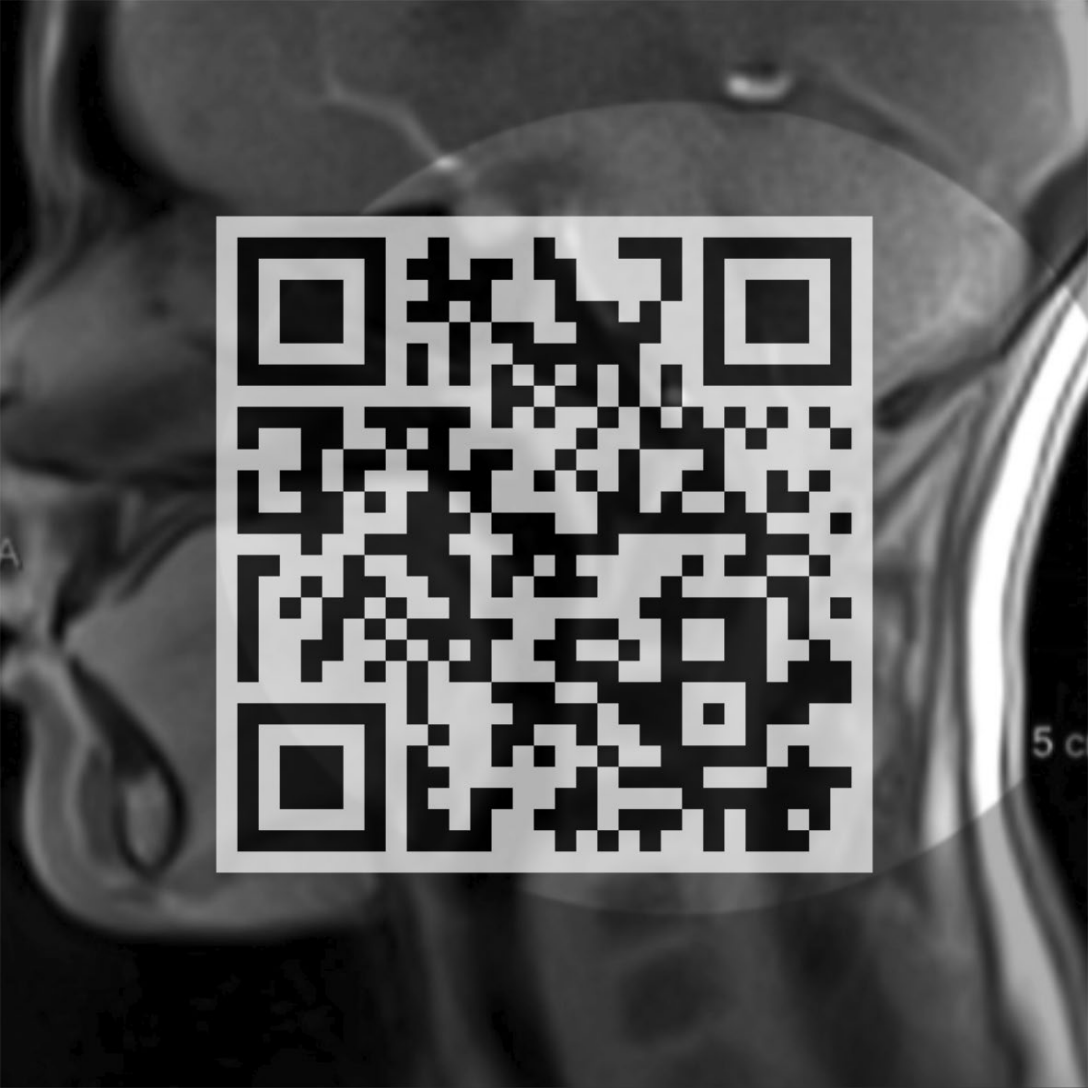
Video 11 Cervical spine study (proton density weighted) in a 12-year-old boy with achondroplasia with a passive movement of the cervical spine by an accompanying person in this non-sedated patient. A slight narrowing of the craniocervical junction is observed during reclination. Please also note the altering signal intensity of the cerebrospinal fluid due to its physiologic motion

**Fig. 2:**
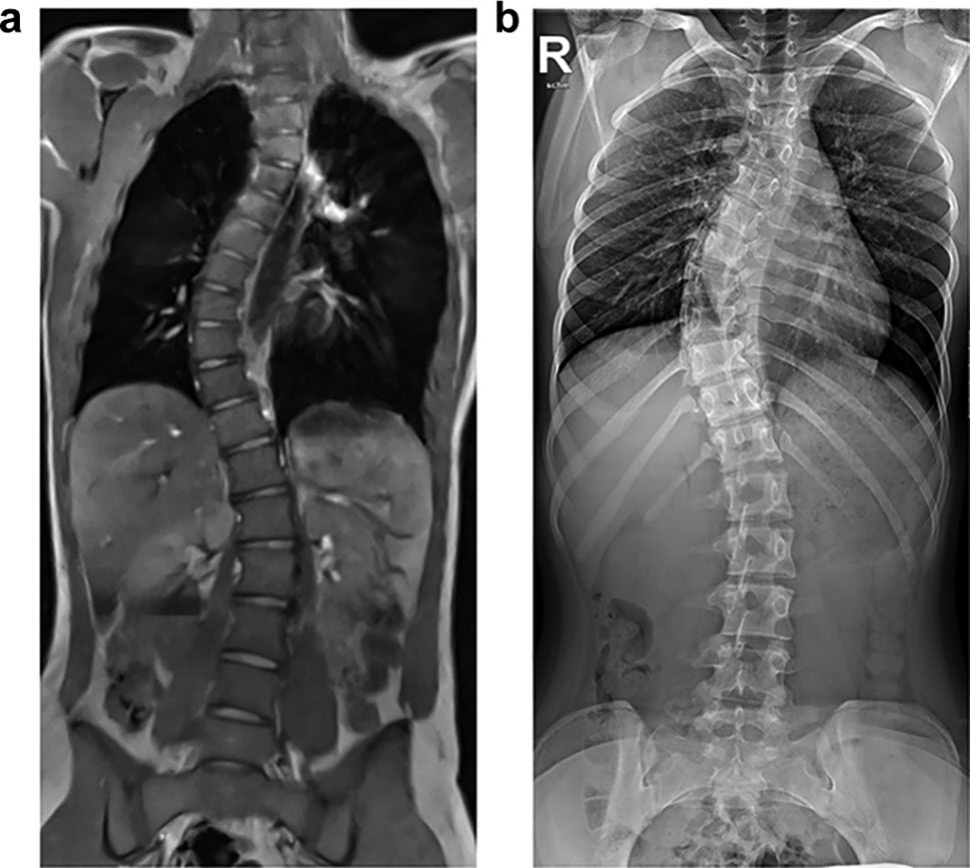
15-year-old girl with juvenile scoliosis. **a** Curved multiplanar reconstruction of a composed volume coverage real-time MRI sequence. **b** Corresponding conventional, stitched X-ray of the spine

In addition to dynamic imaging of the spine, static images of the whole spine can also be generated in a few seconds using VC. Depending on the height of the spine and the available field of view, two to three stacks are needed. Thus, the entire scan takes 40 s, with positioning the patient taking a total of about 6 min. This examination time is in the same range as an X-ray image of the whole spine (unpublished data). The single image stacks are afterward combined to compose an image stack of the whole spine. In post-processing, curved multiplanar reconstructions generate an image impression of a conventional X-ray image in both the anterior–posterior projection and the coronal projection (Fig. 2; Video 12). The Cobb angle and the degree of maturation of the iliac crest are readily assessed in rt-MRI. The omission of an annual x-ray of the whole spine would considerably reduce exposure to ionizing radiation. On the other hand, a conventional, state-of-the-art MRI in every patient with scoliosis would be unnecessary and a waste of resources, as only recently has it been demonstrated that a conventional MRI of the spine and myelon is only required in children that present with neurological symptoms [28].

**Figure:**
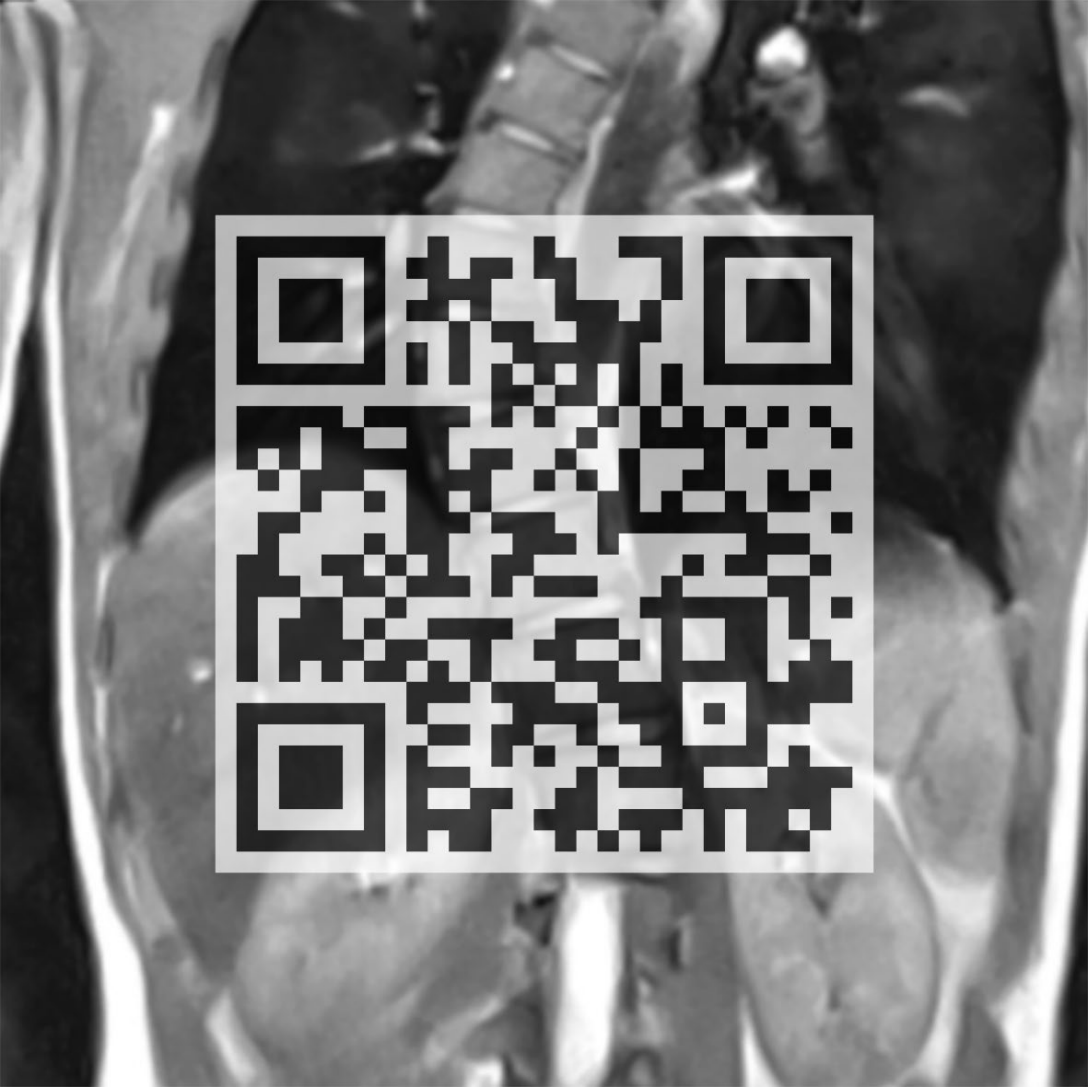
Video 12 T1/T2-weighted volume coverage video of a whole spine in a twelve-year-old boy with scoliosis. The total acquisition time for the three stacks was 40 seconds. Subsequently, a video of the whole spine is composed with a standard MRI stitching tool

## Funnel chest in motion

Real-time MRI is an option to evaluate highly dynamic structures such as the chest wall in patients with pectus excavatum [29]. Specific patterns of the chest wall motion might be of prognostic value. In addition to the presurgical assessment of the chest morphometry, a non-invasive method for correction of pectus excavatum, the vacuum bell, can be directly monitored in rt-MRI. The maximal results to expect after several years of therapy with a vacuum bell can be directly visualized. Furthermore, possible cardiac and pulmonary decompression can be monitored (Video 13).

**Figure:**
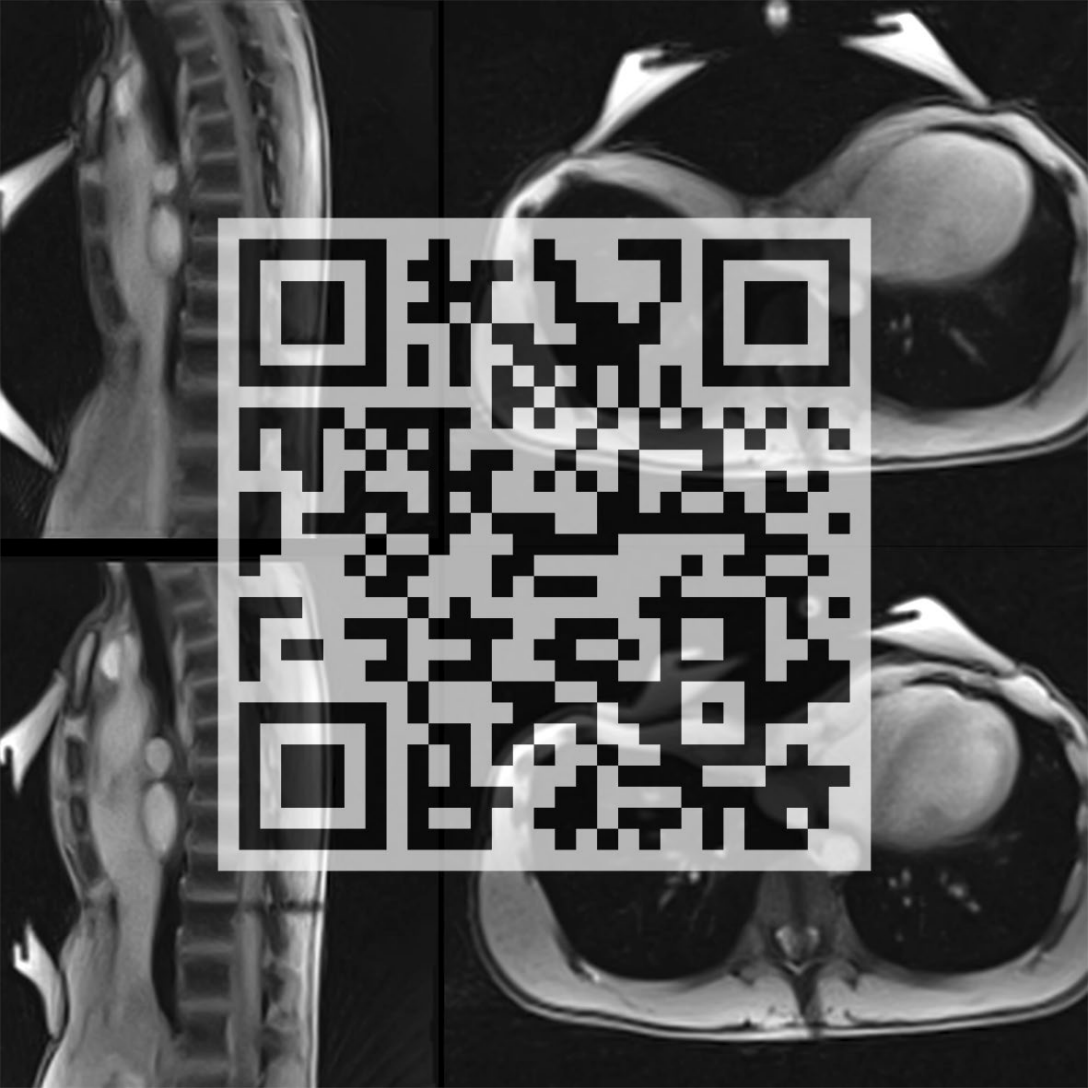
Video 13 16-year-old patient with asymmetric funnel chest. An axial and a sagittal plane are acquired simultaneously in proton density weighting (upper images). Additionally, a non-surgical therapy with a vacuum bell, which lifts the anterior chest wall, was simulated (lower images)

## Real-time MRI of the abdomen

The third option of rt-MRI, interactive navigation, proves useful in abdominal imaging. It allows navigating orientation under permanent visualization. This enables, for example, imaging of bowel movements with and without endoluminal contrast medium, depiction of the ureter and its peristalsis, and defecography in status (Video 14). Yet, there are no reports in this area so far, neither in adults nor in children. Further research is clearly warranted. This technique is most likely to be applied in pediatric interventional radiology.

**Figure:**
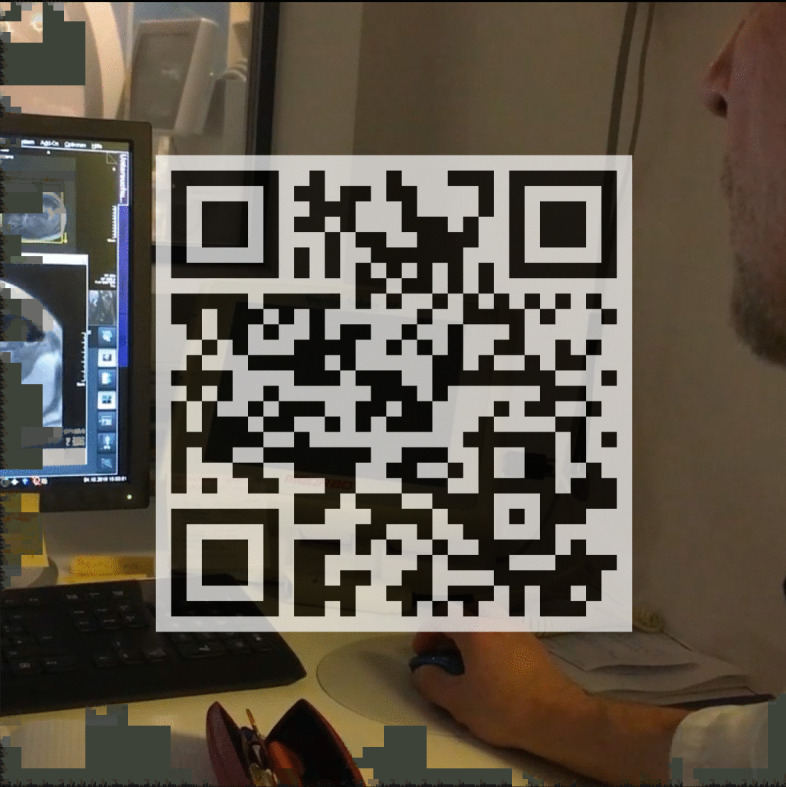
Video 14 Video of online navigation (proton density weighted) at the MR console to visualize the ureter in a 6-year-old child with renal pelvic congestion. Each plane can be chosen as needed in real-time. When altering the plane, there is only a very short drop in the signal intensity until the steady state of the proton excitation is reached again

## Summary

Rt-MRI is a novel imaging modality that seems tailored for children. Due to the ultrafast frame rate, macro-movements, as well as physiological movements of children’s organs, are frozen. This renders rt-MRI a viable option for imaging awake infants and toddlers without sedation, regardless of a suitable US-window.

Due to the different image appearance and the lower spatial resolution compared to conventional MRI, rt-MRI competes more with US diagnostics than with standard MRI in most indications. In departments with the possibility of rt-MRI, ultra-fast examinations of the brain in a few seconds without motion artifacts have become clinical standards and significantly reduced sedation rates. In addition, new indications are emerging, such as imaging of the lungs and the spine, as well as dynamic examinations of thoracic wall malformations or the soft palate before nasopharyngeal insufficiency.

### Supplementary Information

Below is the link to the electronic supplementary material.

Video 1 (MP4 9543 KB)

Video 2 (MP4 2947 KB)

Video 3 (MP4 2390 KB)

Video 4 (MP4 2987 KB)

Video 5 (MP4 4718 KB)

Video 6 (MP4 4188 KB)

Video 7 (MP4 6923 KB)

Video 8 (MP4 3842 KB)

Video 9 (MP4 5799 KB)

Video 10 (MP4 4486 KB)

Video 11 (MP4 1790 KB)

Video 12 (MP4 2585 KB)

Video 13 (MP4 5541 KB)

Video 14 (MP4 6819 KB)

## Abbreviations

CSF: Cerebro-spinal fluid
rt-MRI: Real-time MRI
US: Ultrasound
VC: Volume coverage
